# Adjuvant Endocrine Therapy in Breast Cancer: A Novel e-Health Approach in Optimizing Treatment for Seniors (OPTIMUM): A Two-Group Controlled Comparison Pilot Study

**DOI:** 10.2196/resprot.6519

**Published:** 2016-11-07

**Authors:** Ari Meguerditchian, Robyn Tamblyn, Sarkis Meterissian, Susan Law, Jaroslav Prchal, Nancy Winslade, Donna Stern

**Affiliations:** ^1^ Clinical and Health Informatics Research Group McGill University Montreal, QC Canada; ^2^ Department of Surgery McGill University Montreal, QC Canada; ^3^ Department of Oncology McGill University Montreal, QC Canada; ^4^ Breast Clinic McGill University Health Centre Montreal, QC Canada; ^5^ Department of Epidemiology, Biostatistics and Occupational Health McGill University Montreal, QC Canada; ^6^ Department of Medicine McGill University Montreal, QC Canada; ^7^ Department of Family Medicine McGill University Montreal, QC Canada; ^8^ Research Centre St. Mary's Hospital Montreal, QC Canada; ^9^ Department of Oncology St. Mary's Hospital Center Montreal, QC Canada

**Keywords:** administrative claims, health care, breast neoplasms, medical informatics applications, aromatase inhibitors, telemedicine, health services for the aged, medication adherence

## Abstract

**Background:**

In women with hormone receptor positive breast cancer, adjuvant endocrine therapy (AET) is associated with a significant survival advantage. Nonadherence is a particular challenge in older women, even though they stand to benefit the most from AET. Therefore, a novel eHealth tool (OPTIMUM) that integrates real-time analysis of health administrative claims data was developed to provide point-of-care decision support for clinicians.

**Objectives:**

The objectives of the study are to determine the effectiveness of a patient-specific, real-time eHealth alert delivered at point-of-care in reducing rates of AET discontinuation and to understand patient-level factors related to AET discontinuation as well as to assess integration of eHealth alerts regarding deviations from best practices in administration of AET by cancer care teams.

**Methods:**

A prospective, 2-group controlled comparison pilot study will be conducted at 2 urban, McGill University–affiliated hospitals, the Royal Victoria Hospital and St. Mary’s Hospital. A minimum of 43 patients per study arm will be enrolled through site-level allocation. Follow-up is 1.5 years. Health care professionals at the intervention site will have access to the eHealth tool, which will report to them in real-time medical events with known associations to AET discontinuation, an AET adherence monitor, and a discontinuation alert. Cox proportional hazard ratios with 95% confidence intervals will estimate risks of AET discontinuation. Tests for significance will be 2-sided with a significance level of *P*<.05.

**Results:**

This protocol has been approved and funded by the Canadian Institutes of Health Research. The study will evaluate site-level differences between AET discontinuation and AET adherence and assess care team actions at the intervention site. Participant enrollment into this project is expected to start September 2016 with primary data ready to present by June 2018.

**Conclusion:**

This study will offer an opportunity to verify the feasibility of integrating an eHealth tool that aims to improve the long-term management of breast cancer in a high-risk population by allowing more timely intervention to prevent or rapidly address AET discontinuation.

## Introduction

### Antiestrogen Therapy in Seniors With Breast Cancer: An Effective Anticancer Strategy

Adjuvant endocrine therapy (AET) inhibits the estrogenic stimulation that drives breast cancer growth [1-4]. In women with hormone receptor positive breast cancer, AET is associated with a significant survival advantage [2,5-8]. For instance, tamoxifen reduces the relative risk of recurrence and death from breast cancer by 46% and 26%, respectively [6], with maximal benefits attained from at least 5 years of therapy [9-13]. Similar outcomes are reported with aromatase inhibitors [10,14-17]. Therefore, when taken for at least 5 years, AET is a low-risk, easily administered treatment that constitutes an ideal strategy in reducing the impact of disease in women with hormone receptor positive breast cancer.

Defined as the extent to which patients take a medication as prescribed [18-22], adherence is the most important modifiable factor that can potentially compromise treatment outcome [23,24]. Adherence problems lead to worsening of disease, increased hospitalizations and health care costs, and death [25-31]. Suboptimal adherence can be due to factors related to patient, provider, and health care system characteristics [18,21]. Despite the magnitude of breast cancer as a health problem and the impressive survival benefits of AET, nonadherence rates of more than 20% have been noted in tightly controlled clinical trials [32-34]. However this does not accurately reflect the reality of vulnerable patients such as older women, who tend to be underrepresented in research studies [35-38].

Limited studies show that adherence to AET is a particular challenge in older women [39]. Paradoxically, these women stand to benefit the most from AET, because breast cancer is hormone receptor positive in more than 80% of women 65 years and older [40-46], older patients are often precluded from other more toxic forms of systemic treatment such as chemotherapy [47], and the use of AET simplifies managing breast cancer in seniors by eliminating the need for other forms of treatment. For example, radiotherapy can be omitted in patients 70 years and older after breast conserving surgery for stage I disease, providing they receive AET [48,49]. Specifically, in patients older than 65 years old, AET has been shown to improve 15-year survival by at least 21% [50]. Consequently, the International Society of Geriatric Oncology recommends that seniors with hormone receptor positive breast tumors benefit from AET, because there is no evidence of age-related differences in the efficacy of tamoxifen and aromatase inhibitors [50,51].

Because close to 40% of breast cancers in Canada are diagnosed in women 65 years and older and 61% of deaths from this disease occur in women 70 years and older [52], it is crucial that we gain a better understanding of problems associated with AET adherence in this population.

### Adherence to Adjuvant Endocrine Therapy: Tackling Challenges in Older Women

Conventional strategies in documenting adherence to AET (eg, chart review, patient self-report, metabolite measurement, pill counts) are limited by their reliability and applicability [53-57]. They do not correspond well to the reality of taking AET, a self-administered daily treatment that requires minimal interaction with the care team, for seniors who often have other comorbidities (and thus additional prescriptions). As a result, opportunities to identify problems throughout the course of treatment and intervene are limited [58,59].

Generated for the purpose of directing payment, administrative claims constitute a source of potentially complete health care information covering all services provided to a patient, including outpatient drugs [60]. We have previously demonstrated that pharmacy claims were superior to a national cancer registry in reporting delivery of outpatient self-administered treatments. Initiation of AET was documented in an additional 55% of patients in comparison to registry data from the National Cancer Data Base [61].

Online adjudication processes of drug insurance programs and electronic storage of claims data ensure both timeliness and longitudinal compilation of information on drug utilization. This can be used to assess adherence to AET by seniors by calculating the medication possession ratio (MPR) based on refill rates, which reflect the availability of medication supply [62]. Defined as the ratio of total days covered by medication divided by the number of days needing the medication [21,63], the MPR estimates the proportion of days on which medication is taken and appears to be a better predictor of therapeutic outcomes compared to self-report or pill count [64-66].

Using pharmacy claims from all Quebec breast cancer patients (1998-2007), we have shown that adherence to AET among older women is suboptimal across each of the 5 years of treatment, falling below 75% at the fifth year [67,68]. We have shown that 37% of patients experience discontinuation of some duration within the first year [67]. Using health service claims, we have further demonstrated that hospitalizations, addition of new drugs during therapy, switch in AET type (tamoxifen versus aromatase inhibitor), and depression are associated with higher rates of nonpersistence.

To date, very few studies have used pharmacy claims to characterize AET adherence in seniors with breast cancer. Using the cohort of Quebec women aged 70 years and older (1998-2005), our group has demonstrated that 32% of older patients discontinued AET at some point during therapy and 20% of these women permanently abandoned therapy, thus losing the significant age-specific survival advantage provided by this treatment [50,69,70]. We have also shown that treatment discontinuation was more frequent in seniors who had encountered irregularities and gaps in quality in other aspects of breast cancer care.

### Medical Office of the XXIst Century: A Novel eHealth Tool to Optimize Breast Cancer Care Delivery in Seniors

Medical Office of the XXIst Century (MOXXI) is a novel clinical informatics system that provides patient-specific, point-of-care documentation and decision support through real-time processing of health services claims [71-73]. This platform also allows analysis of treatment plan variations. The disease-specific, real-time, point-of-care informatics support provided by MOXXI has been associated with better, safer care. For example, in the case of cardiovascular medications, physicians who received automated alerts from MOXXI regarding low treatment adherence (based on a real-time feed from the provincial pharmacy claims database) were significantly more likely to review the patient’s drug profile and take appropriate action [73]. Another MOXXI application directed at older adults provides real-time alerts to physicians regarding the risk of fall-related injury in relation to the patient’s psychoactive medications. This eHealth tool has been shown to result in treatment plan modifications in 24.6% of patients and a reduction of injury risk by 1.7 injuries per 1000 patients [74,75].

The integration of eHealth tools such as MOXXI represents a unique opportunity to address challenges in the delivery of care for older women diagnosed with breast cancer [76]. With multiple medical comorbidities, extensive health service use, and frequent deviations from best practices for cancer treatment, this group stands to benefit the most from the development of a comprehensive cancer care quality strategy addressing all components of treatment, continuously integrating all aspects of their health, and supporting treatment decisions specific to their needs [77,78].

### Purpose and Objectives

The aim of this study is to verify if a patient-specific eHealth tool (OPTIMUM) that integrates real-time analysis of administrative claims data and provides point-of-care risk assessment to care teams will optimize breast cancer treatment by increasing adherence and persistence to antiestrogen therapy in patients aged 65 years and older.

The first objective is to determine the effectiveness of OPTIMUM’s patient-specific, real-time eHealth alerts in reducing rates of AET discontinuation in older women with breast cancer and understand patient-level factors related to AET discontinuation.

The second objective is to assess integration of the eHealth alerts reporting deviations from best practices in administration of AET by evaluating cancer care team responses.

## Methods

### Overview

**Figure 1:**
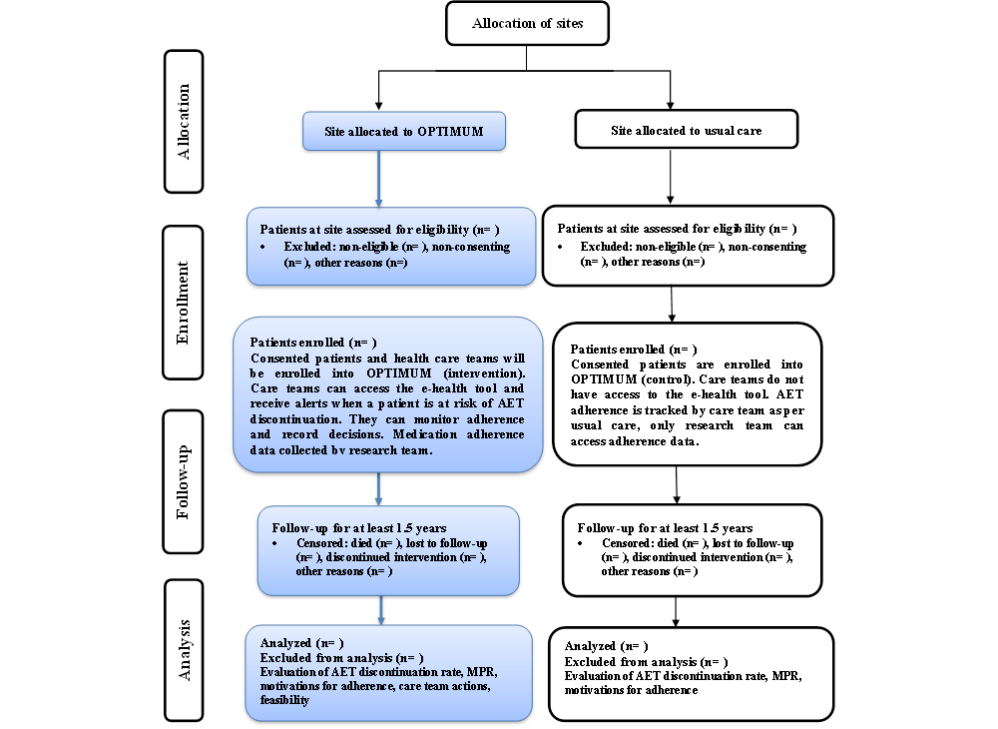
Flowchart of study design: a prospective intervention study with a contemporaneous control group.

A prospective, 2-group controlled comparison pilot study will be conducted to test the hypothesized benefits of the OPTIMUM eHealth tool with integrated alerts in managing AET in seniors with breast cancer (see Figure 1 for study flow). The benefits of the intervention will be assessed by comparing patients of care teams using the OPTIMUM eHealth tool with patients of care teams contemporaneously provided usual care. The follow-up will last 1.5 years based on previous findings by our group that the median time to first AET discontinuation in 3180 Quebec women (aged 70 years and older) with early stage breast cancer was 1.5 (range 0.5-3.4) years [69]. Ethics approval will be obtained before initiating the study.

### Study Population and Setting

The study will take place at 2 urban hospitals in Montreal, Canada: the Royal Victoria Hospital (RVH) and St. Mary’s Hospital (SMH). These hospitals were selected for comparable resources and treatment standards. Both hospitals are affiliated with McGill University and benefit from its clinical trials infrastructure. In addition, both sites have similar multidisciplinary care teams consisting of surgical, medical, and radiation oncologists and an oncology nurse specialist. Staff and support resources are allocated at the institution level, where patient management is standardized. A total of 1168 new breast cancer patients were treated at both sites in 2012 with about 40% of the women being over the age of 65 years [79].

### Eligibility Criteria

Eligible patients from the 2 study sites will be identified by the oncology nurse specialist. To be eligible to participate in this study, patients must be 65 years or older, able to give consent, and diagnosed with incident (nonmetastatic) breast cancer after having undergone breast surgery for stages I-III disease. Patients must have had medical insurance with the Régie de l’Assurance Maladie du Québec (RAMQ) for at least 1 year prior to surgery and have histologically confirmed breast adenocarcinoma with hormone receptor positive disease. Patients must have no history of AET use prior to the diagnosis of breast cancer, expect to initiate AET or have only recently initiated AET (less than 6 months), and be free of previous discontinuation events.

### Recruitment

Patients will be recruited exclusively from the breast centers participating in the study (RVH and SMH). Advertisements will be placed in the registration areas, waiting rooms, and clinical care areas. Care teams (physicians, oncology nurses, and rehabilitation specialists) will facilitate identification of eligible patients and secure their permission to be approached by a study coordinator who will inform patients about the study and provide a detailing pamphlet. Only patients and health care professionals who have provided a standardized consent form with their signatures will be enrolled in the study. Both patients and care teams will be aware that the intervention site will receive the eHealth tool to test the study hypothesis. However, only the health care professionals will know if their site has the intervention based on their site’s receipt of the eHealth tool. This intervention is not expected to impact the care of patients at the control site because AET adherence optimization is part of best practices. For additional validation, adherence of participants at the control site can be compared to historical population-based adherence data [67,68].

### Intervention Allocation

All eligible patients who provide consent will be enrolled into OPTIMUM. The intervention allocation will occur at the site level. The care team at RVH will have access to the OPTIMUM eHealth tool (intervention). At SMH, the care team will continue to deliver care according to standard processes (control group).

The control and intervention sites were selected for their similarities in source population, breast cancer volume, practice profiles, and academic affiliation. In addition, both institutions recently underwent administrative and physical reorganizations of the same magnitude. RVH completed their transition several months earlier than SMH, which enabled the process of logistical implementations related to the intervention and ethics approval for this pilot to start earlier there.

### Intervention: An eHealth Tool

**Figure 2:**
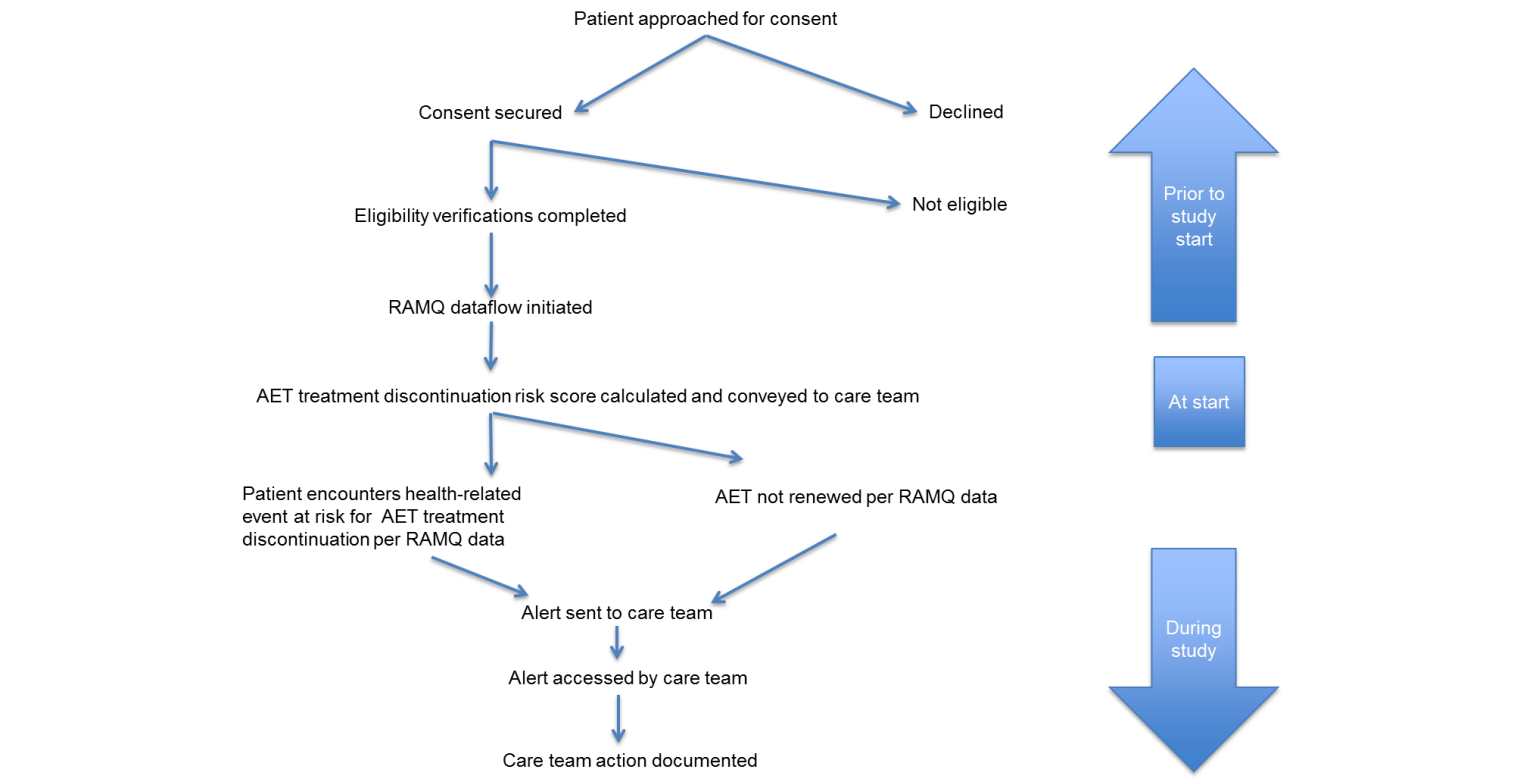
Process flow for care teams.

The intervention site care team will receive the following 3 OPTIMUM eHealth alerts (for care team process flow, see Figure 2).

#### Electronic Alert of Increased Adjuvant Endocrine Therapy Discontinuation Risk

Through real-time analysis of health service claims, OPTIMUM will generate this alert when a hospitalization, emergency room visit, addition of new medications, or change in AET drug occurs (see Multimedia Appendix 1-2 for examples of alerts from another eHealth tool trial). We have previously demonstrated that the occurrence of these during AET negatively impacts treatment adherence. The care team will be prompted by the eHealth tool to log its actions upon receipt of these alerts. The choice of actions is at the discretion of the care team and may include performing telephone follow-up with patient, performing telephone follow-up with community pharmacist, arranging for return to clinic with doctor, arranging for return to clinic with nurse, or ignoring the alert (see Multimedia Appendix 1-3 for examples of another eHealth tool and Multimedia Appendix 4 for the types of actions the care teams can select when faced with an alert).

#### Adherence Monitor

As the treatment progresses, OPTIMUM will provide a graphic representation of adherence to AET over time for each patient (see Multimedia Appendix 3 for example).

#### Electronic Discontinuation Occurrence Alert

Through real-time analysis of pharmacy claims including information on renewal date and number of pills dispensed, OPTIMUM will generate an electronic alert when there is a gap in AET prescription renewal within 10 days of the expected date. This threshold was determined from secondary analysis of our previous publications [67,68], which found that most patients who did not discontinue permanently renewed AET within 10 days. Again, the care team will be prompted by the eHealth tool to log its actions upon receipt of these alerts. The choice of actions are at the discretion of the care team and may include performing telephone follow-up with patient, performing telephone follow-up with community pharmacist, arranging for return to clinic with doctor, arranging for return to clinic with nurse, or ignoring the alert (see Multimedia Appendix 1-3 for examples of another eHealth tool and Multimedia Appendix 4 for the types of actions the care teams can select when faced with an alert).

### Control Site

Care teams at the hospital assigned as control site will follow up with patients according to standard processes of care and will not have access to the eHealth tool.

### Sample Size

A minimum of 120 patients will be approached to obtain a pilot sample size of 43 patients per arm [80,81]. This assumes comparable care standards between arms and among clinicians, an enrollment rate of 75%, a 5% loss to follow-up for infrequent cases of patient migration or opting out of the government drug plan [68], with a proportion of 20% permanently discontinuing at the control site [67,68] and 14% in the intervention group (minimally clinical important risk difference of 6%, a relative effect of 30%).

### Data Sources

The following data sources will be linked to obtain important patient, disease, and clinical information and verify study outcomes (see Textbox 1).

Registrant database of insured persons documents year of birth, gender, 3-digit postal code, and date of death for all Quebec residents eligible for provincial health insurance coverage (approximately 99% of the population of Quebec), as well as dates for any noninsured periods [82,83].

RAMQ medical service claims database (RAMQ-MD) contains records for all services provided within Quebec’s public health insurance plan by physicians remunerated under the fee-for-service system (approximately 96% of physicians in Quebec). This database includes encrypted physician license number, physician speciality, service date, code for the service provided, location of service delivery (eg, community health services center, hospital) and primary diagnosis codes (*International Classification of Diseases, Ninth Revision* [ICD-9]). Previous studies have demonstrated that diagnostic codes in medical service claims have high specificity, high positive predictive value, and high negative predictive value, estimated at above 90% for all 3 indicators [84].

RAMQ drug insurance eligibility database contains start and end dates of patient eligibility for public drug insurance as well as the type of drug plan.

RAMQ prescription claims database (RAMQ-Rx) contains claims for prescription drugs dispensed to all Quebec residents insured under the public drug plan. It includes encrypted physician license number, Drug Identification Number of drug dispensed, date the medication was dispensed, quantity dispensed, and duration of the prescription. Our group has shown that the accuracy of this database in identifying the correct drug dispensed is over 99% [85].

MedEcho is the hospital’s discharge database, which includes admission date, principal and secondary diagnoses, and services performed for all discharges from acute care hospitals in the province. The first hospital-based service for breast cancer is considered the patient’s index date.

Hospital chart includes all clinical notes of treatment decisions and clinical course.

The RAMQ links the above data sources using the *numéro d’assurance-maladie*, a unique identifier attributed to each Quebec resident and common to these databases. Appropriate clearance has been obtained from the *Commission d’accès à l’information du Québec* for the use of these population databases.

**Data sources and look-back periods with type of information extracted.**

Registrant database of insured persons—12 month prior to index date

- Date of Birth
- Health insurance eligibility status

RAMQ medical service claims database (RAMQ-MD)—24 months prior to index date

- Validation of index date
- Medical services received

Drug insurance eligibility database—12 months prior to index date

- Verification of coverage eligibility

RAMQ prescription claims database (RAMQ-Rx)—12 months prior to index date

- Drugs received
- Polypharmacy

MedEcho—12 months prior to index date

- Index date ascertainment
- Admissions
- Discharges
- Emergency department visits

### Confounding Variables

#### Patient Demographics (Fixed Covariates)

Age at diagnosis will be obtained from the registrant database. Socioeconomic status information will be obtained using the RAMQ-Rx database. A variable will be created grouping patients according to income supplementation received by the government. Patients will either “not qualify for a supplement,” “qualify for some supplement,” or “qualify for maximum supplement.”

#### Disease Characteristics (Fixed Covariate)

We will identify the patient’s breast cancer stage using topography and morphology (ICD-9) codes recorded in the hospital’s discharge database (MedEcho).

#### Treatment Characteristics (Fixed Covariates)

We will characterize delivery and date for each component of breast cancer care (itemized below) using medical services billing codes in the RAMQ-MD and prescription drug claims in the RAMQ-Rx databases. As previously discussed, claims offer the opportunity to accurately monitor breast cancer patient progress through the cancer care continuum because they cover all services provided regardless of practitioner or site.

- Breast surgery: mastectomy, lumpectomy, no surgery
- Axillary surgery: sentinel lymph node biopsy, axillary lymph node dissection, no lymph node surgery
- Radiation therapy: consultation with radiation oncology, delivery of external beam radiation therapy, no radiation therapy

Systemic therapy: consultation with medical oncology, delivery of systemic chemotherapy, no chemotherapy

AET initiation: 3 variables will be created—choice of drug (tamoxifen, anastrosole, letrosole or exemestane), timeliness of treatment initation (1 year or less), and whether the patient switched type of AET during follow-up

#### Other Medical Conditions (Fixed Covariates)

Romano’s adaptation of the Charlson comorbidity index will be used to measure each patient’s baseline risk of discontinuing in relationship to their health status using ICD-9 codes listed in claims in the 12 months prior to the diagnosis of breast cancer. In addition, the MedEcho database will be used to document the number of emergency department visits and inpatient admissions for each 12-month period following AET start.

#### Polypharmacy (Fixed Covariates)

Drug claims for the 12 months preceding AET start, supplied by the RAMQ-Rx, will be classified according to the American Hospital Formulary Classification to determine the baseline number of different categories of non-AET drugs prescribed at the start of the AET treatment.

#### Physician Characteristics (Fixed Covariates)

Prescribing physician specialty, supplied by the RAMQ-Rx, may influence a patient’s adherence to treatment. Therefore, the specialty of the first physician to prescribe AET to the patient will be identified. We will calculate the number of women with a breast cancer diagnosis that this first prescribing physician billed a medical service claim for in the year prior to initiating the patient’s treatment. In addition, the physician’s experience in treating breast cancer patients will be obtained. Finally, the number of different physicians who prescribed the patient’s AET will be recorded. No information that can potentially identify physicians will be collected.

### Definitions of Outcomes

The primary outcome is an AET discontinuation event defined as failure to refill an AET prescription within 10 days after the due refill date. The first due refill day preceding a gap of 10 days would be considered the date of discontinuation event. Permanent AET discontinuation will be defined as failure to refill an AET prescription before the end of the first year of treatment. First-year adherence will be measured by the MPR, defined as the proportion of days covered by medication supply in the treatment period. Actions taken by care teams upon receipt of an alert from the OPTIMUM eHealth tool will be observed and logged with the purpose of developing future AET adherence intervention strategies. These actions could be part of a drop-down menu and may include: perform telephone follow-up with patient, perform telephone follow-up with community pharmacist, arrange for return to clinic with doctor, arrange for return to clinic with nurse, or ignore the alert.

### Operationalization of Outcomes

**Figure 3:**
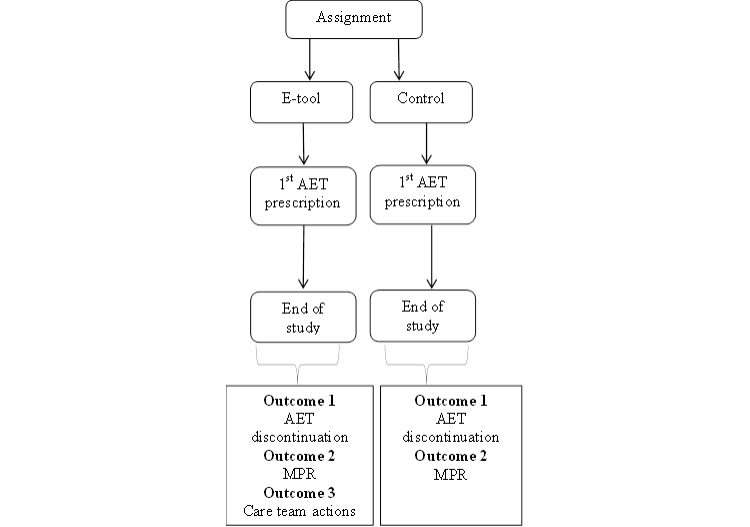
Outcomes by study arm.

Visual representation of outcomes to be quantified during the course of the study and according to the study arm is shown in Figure 3.

AET discontinuation will be assessed by determining the proportion of patients permanently discontinuing AET treatment, proportion of patients reinitiating after a discontinuation of AET treatment, and the mean time to AET treatment reinitiation. This will be calculated in both the intervention and control arms. These outcomes will then be compared between both arms at the end of the study by the research team.

The MPR will be assessed as the mean MPR, and the proportion of patients that maintain an MPR of 80% or more. This will calculated in both intervention and control arms. Outcomes will be compared between both arms at the end of the study by the research team.

Types of actions taken by health care teams upon alert receipt will be assessed qualitatively and quantitatively in the intervention arm. As detailed above, the eHealth tool users will be prompted to document actions in response to an alert through a drop-down function in OPTIMUM. These actions will be logged in real-time when a notification is accessed and recorded. The outcome will be tabulated at the end of the study by the research team.

### Statistical Analysis

#### Assessing Effectiveness of the OPTIMUM eHealth Tool at Reducing Adjuvant Endocrine Therapy Rates of Discontinuation

In order to assess if the OPTIMUM eHealth tool has an impact on adherence, rates of AET discontinuation and MPR will be evaluated and compared between the intervention and control groups at the end of the follow-up period. Univariate and multivariate Cox proportional hazards regression models will be used to analyze the association between rates of AET discontinuation in patients managed with the OPTIMUM eHealth tool versus those in usual care, while adjusting for known patient clinical and demographic factors. These factors include age, hospitalizations, type of AET, depression, Charlson Comorbidity Index, and polypharmacy at baseline [69].

Time to treatment discontinuation will be measured using Kaplan-Meier analysis. The MPR will be calculated at the end of the study period. Patients will be classified as adherent if they maintain an MPR of 80% or more. Univariate and multivariate logistic regression analysis will be used to assess the association between the OPTIMUM eHealth tool and maintaining an MPR of 80% of more while adjusting for known risk factors. The proportion of patients discontinuing in each group (intervention vs usual care) and restarting medication will be compared using a chi-square test. The mean time to restart will be compared using a *t* test in those who restart after a treatment discontinuation event.

#### Assessing Integration of eHealth Alerts Concerning Deviations From Best Practices

Actions taken by health care teams in response to alerts (intervention arm) will be documented and assessed both qualitatively and via descriptive statistics. Descriptive statistics will be used to evaluate the most common action performed upon receiving an alert, frequency of log-ins to visit the patient’s profile, frequency of viewing the adherence monitor, frequency and types of actions selected from the drop-down menu, or other actions such as change in treatment plan.

### Confidentiality

#### Identity Protection

In order to protect confidentiality for study participants, all identifying information will be removed from the files prior to analysis. Therefore, research data transactions will be performed on deidentified data only using a unique study number assigned to each subject (includes patients and physicians). Only this number will be used on research documents that relate to the subject. Team members will be required to sign a confidentiality agreement with the principal investigator prior to accessing the information. The linkage table of subject names and corresponding study numbers will be kept in a secure locked location accessible only to the principal investigator (or his delegate). No names will be released and only grouped data will be presented in oral or written scientific communications. Identifying information will be destroyed 5 years after publication of findings.

#### Data Storage

Collected research data will be saved on research servers independent from and outside of the 2 clinical care sites. These servers are located at McGill University’s data center, and access to them is protected by alarm system and 24-hour guard surveillance. In addition, informatics data transactions through these servers occur in the context of a private network and require username and password. Transactions are recorded and trackable by our in-house information technology networks specialist. Access to the server is restricted; it has the latest and most sophisticated protection against unauthorized intrusions and potential damage. This type of design offers a high level of data security in the event that a computer is lost or stolen.

### Ethical Considerations

The OPTIMUM study will be conducted according to ethical principles stated in the Declaration of Helsinki (2013). This study received ethics approval from McGill University Health Centre Research Ethics Board on August 16, 2016. Consent forms will take into consideration the well-being, free-will, and respect of the participants (including respect of their privacy).

## Results

Participant enrollment into this project is expected to start in September 2016 with primary data ready to present by June 2018.

## Discussion

Over 40% of breast cancers in Canada occur in women 65 years and older. AET is an effective approach in reducing recurrence and cancer death in these women. Unfortunately, adherence to AET in this population is suboptimal.

This study aims to use a 2-group controlled comparison pilot study to verify the feasibility of integrating eHealth tools that aim to improve the management of the breast cancer in this high-risk population by allowing more timely intervention to prevent or rapidly address treatment discontinuation. Our group has already developed a successful model of using information technology to provide real-time decision support and feedback to care teams on patient-specific risk profiles. With the knowledge generated from this research, we plan to develop an informatics tool that would provide health professionals with a new generation of decision-support tools specific to the older cancer patients’ needs. As this study is a pilot, feedback from care teams on ease of integrating the eHealth tool into existing workflows will support and guide the launch of a larger scale longitudinal study.

Our multidisciplinary team of scientists brings together research expertise from medicine, surgery, nursing, pharmacy, psychology, and evaluative sciences in developing the next generation of informatics-enabled tools in optimizing breast cancer care. Research will take place at the Clinical and Health Informatics Research Group. The Group is experienced in handling electronic clinical data systems such as hospital clinical data warehouses, RAMQ administrative databases, and clinical competency data through the Medical Council of Canada/College of Physicians of Quebec. Over the past decade, it has developed extensive know-how in developing value-added health informatics tools to improve health outcomes.

## Appendix

Multimedia Appendix 1 Visual depiction of the type of patient-specific alerts that can be provided to the health care professional borrowed from another trial on the effectiveness of MOXXI in improving care quality. This is an example of a patient-specific, real-time MOXXI alert provided to the care team regarding a prescription duplication and specific drug-related risk.

Multimedia Appendix 2 Visual depiction of the type of patient-specific alerts that can be provided to the health care professional borrowed from another trial on the effectiveness of MOXXI in improving care quality. This is a notification of events associated with an increased risk of drug-related issue (ie, emergency department visits).

Multimedia Appendix 3 Visual depiction of the type of patient-specific alerts that can be provided to the health care professional borrowed from another trial on the effectiveness of MOXXI in improving care quality. This is a graphical representation of drug adherence over time.

Multimedia Appendix 4 Example of how MOXXI, an eHealth tool, can be used to document an action and its rationale upon receipt of a patient-specific alert. In this example, the action is to stop a drug, and the drop-down list follows with reasons for changing the prescription.

Multimedia Appendix 6 Protocol approval and review by the Canadian Institutes of Health Research.

## Abbreviations

AET: adjuvant endocrine therapy
ICD-9: International Classification of Diseases, Ninth Revision
MPR: medication possession ratio
MOXXI: Medical Office of the XXIst Century
RAMQ: Régie de l’Assurance Maladie du Québec
RAMQ-MD: RAMQ medical service claims database
RAMQ-Rx: RAMQ prescription claims database
RVH: Royal Victoria Hospital
SMH: St. Mary’s Hospital
