# Supplementary figures and images for: Adjuvant Endocrine Therapy in Breast Cancer: A Novel e-Health Approach in Optimizing Treatment for Seniors (OPTIMUM): A Two-Group Controlled Comparison Pilot Study
Source: JMIR Res Protoc. 2016 Nov 7;5(4):e199. doi: 10.2196/resprot.6519 (PMC5118585; doi:10.2196/resprot.6519)

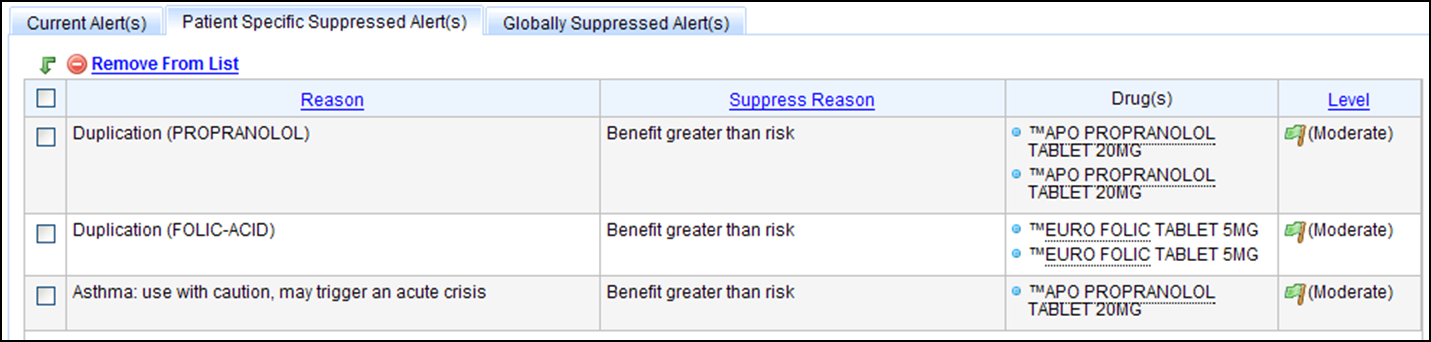

Supplement: Multimedia Appendix 1 [file resprot_v5i4e199_app1.png]

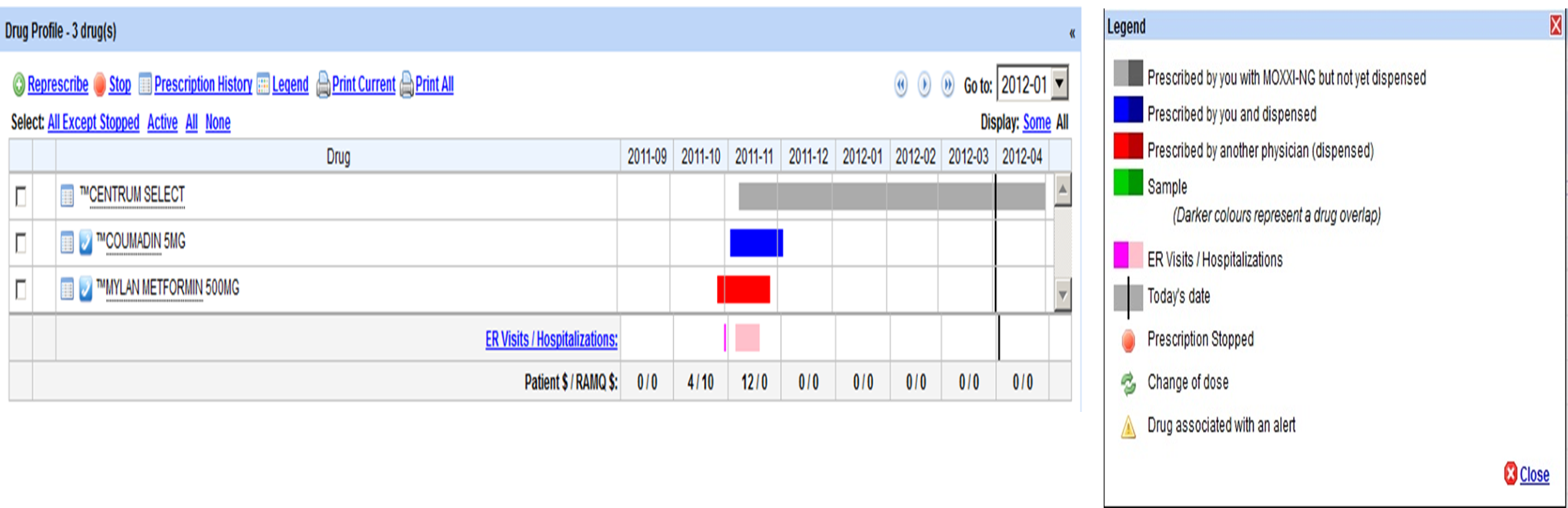

Supplement: Multimedia Appendix 2 [file resprot_v5i4e199_app2.png]

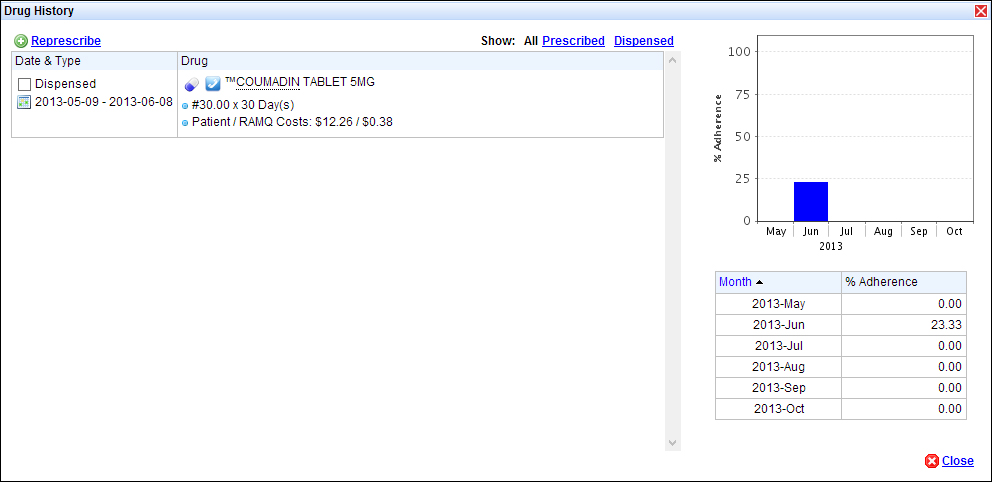

Supplement: Multimedia Appendix 3 [file resprot_v5i4e199_app3.png]

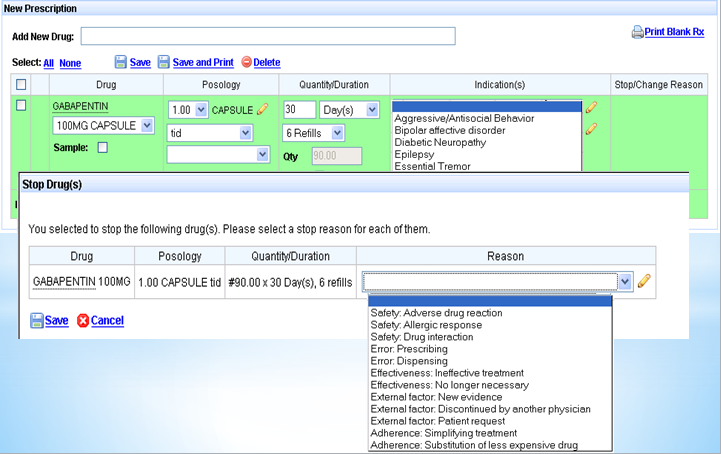

Supplement: Multimedia Appendix 4 [file resprot_v5i4e199_app4.png]
