# Supplementary material for: Adjuvant Endocrine Therapy in Breast Cancer: A Novel e-Health Approach in Optimizing Treatment for Seniors (OPTIMUM): A Two-Group Controlled Comparison Pilot Study
Source: JMIR Res Protoc. 2016 Nov 7;5(4):e199. doi: 10.2196/resprot.6519 (PMC5118585; doi:10.2196/resprot.6519)
Supplement: Multimedia Appendix 6 [file resprot_v5i4e199_app6.pdf]

February/Février 21, 2014

DC0190GP

Canadian Institutes of Health Research / Instituts de recherche en santé du Canada

Notice of Decision / Avis de décision

Application Number/Numéro de la demande: 316814

Committee Code/Code du comité: EHI

Applicants/Candidats: Dr. Ari Nareg MEGUERDITCHIAN

With/Avec: Mrs. J. MARTIN

Institution paid/ Établissement payé: McGill University

Title/Titre: Adjuvant Endocrine Therapy in Breast Cancer: A Novel E-health Approach in Optimizing Treatment Adherence in Seniors

Primary Inst./ Inst. principal: Aging

Other Related Inst./ Autres inst. connexes: Health Services and Policy Research; Cancer Research

Competition Outcome/Résultats du concours: Catalyst Grant: eHealth Innovations October/Octobre 15, 2013

Number in competition/Nbre de demandes dans le concours: 88

Number approved/Nbre de demandes approuvées: 12

Decision on your application/ Décision sur votre demande: Approved Other offer

Average annual amount/ Montant annuel moyen: $0

Equipment amount/ Montant pour les appareils: $0

Term/Durée: 1 **yrs/ans** 0 **months/mois**

Peer Review Committee Recommendation, for your information and use/ Recommandation du comité d'examen par les pairs, pour fins d'information et d'utilisation:

Committee/Comité: e-Health Innovations

Application rank within the competition/ Rang de la demande dans ce concours: 10

Percent Rank Within the Competition/ Rang en pourcentage au sein du concours: 11.36%

Rating/ Cote: 3.95

Recommended average annual amount/ Montant annuel moyen recommandé: $76,336

Recommended equipment amount/ Montant recommandé pour les appareils: $0

Additional Funding Opportunities/ Opportunités de financement additionnelles	Decision/ Décision	Competition Code/ Cote de concours	Application Number/ Numéro de la demande
Catalyst Grant: eHealth Innovations - Aging	Approved	201310CEA	327788

*** Applications receiving a score of less than 3.5 on any evaluation criteria will not be considered for Funding. / Les demandes qui ont reçu une note inférieure à 3.5 pour n'importe quel des critères d'évaluation ne sont pas admissibles.

This document is for information only. Official payment is stated on the CIHR Authorization for Funding.
Document à titre d'information seulement. Le paiement officiel est indiqué sur l'autorisation de financement des IRSC.

CIHR IRSC

Institute of Aboriginal Peoples' Health
Institute of Aging
Institute of Cancer Research
Institute of Circulatory and Respiratory Health
Institute of Gender and Health
Institute of Genetics
Institute of Health Services and Policy Research
Institute of Human Development and Child and Youth Health
Institute of Infection and Immunity
Institute of Musculoskeletal Health and Arthritis
Institute of Neurosciences, Mental Health and Addiction
Institute of Nutrition, Metabolism and Diabetes
Institute of Population and Public Health

Institut de la santé des Autochtones
Institut du vieillissement
Institut du cancer
Institut de la santé circulatoire et respiratoire
Institut de la santé des femmes et des hommes
Institut de génétique
Institut des services et des politiques de la santé
Institut du développement et de la santé des enfants et des adolescents
Institut des maladies infectieuses et immunitaires
Institut de l'appareil locomoteur et de l'arthrite
Institut des neurosciences, de la santé mentale et des toxicomanies
Institut de la nutrition, du métabolisme et du diabète
Institut de la santé publique et des populations

March 15, 2014

Dr. Ari Nareg MEGUERDITCHIAN
McGill University Health Centre
Royal Victoria Hospital
687 Pine Ave. West
Room S7.30
Montreal, Québec H3A 1A1

Dear Dr. MEGUERDITCHIAN:

We are pleased to inform you that the Canadian Institutes of Health Research (CIHR) has approved your recent application entitled "Adjuvant Endocrine Therapy in Breast Cancer: A Novel E-health Approach in Optimizing Treatment Adherence in Seniors". Since you are receiving this letter through ResearchNet your Authorization for Funding will follow in the mail. If you cannot access the review documents related to your proposal through ResearchNet, please contact the CIHR staff member listed below.

Please note that in addition to any budget cuts made by the peer review committee, an additional across-the-board administrative cut of approximately 7.76% was applied to the approved applications to maximize the number of applications funded.

Should you have any questions about the review process, please address them directly to CIHR staff. Do not contact the officers or members of the peer review committee. As CIHR does not notify co-applicants of the decision, we ask that you inform those individuals involved, along with their research institutions (if different from your own), of the outcome of this application.

If you have any questions, please contact Jeff Warren, Program Delivery Coordinator, at 613-948-2813 or jeff.warren@cihr-irsc.gc.ca.

Congratulations on your success in this competition!

Mary Fraser Valiquette

Mary Fraser Valiquette
A/Deputy Director, Program Delivery
Strategic Program Design and Analytics
Research and Knowledge Translation Portfolio

360134-201310CEA-EHI-327788-I007-168217-NCAA2

Canadian Institutes of Health Research
Room 97, 160 Elgin Steet, Address locator: 4809A
Ottawa, (Ontario) K1A 0W9 Tel.: (613) 941-2672
Fax (613) 954-1800 **www.cihr-irsc.gc.ca**

Instituts de recherche en santé du Canada
Pièce 97, 160 rue Elgin, Indice de l'adresse: 4809A
Ottawa, (Ontario) K1A 0W9 Tél.: (613) 941-2672
Fax (613) 954-1800 **www.irsc-cihr.gc.ca**

Canada

CIHR IRSC

Institute of Aboriginal Peoples' Health
Institute of Aging
Institute of Cancer Research
Institute of Circulatory and Respiratory Health
Institute of Gender and Health
Institute of Genetics
Institute of Health Services and Policy Research
Institute of Human Development and Child and Youth Health
Institute of Infection and Immunity
Institute of Musculoskeletal Health and Arthritis
Institute of Neurosciences, Mental Health and Addiction
Institute of Nutrition, Metabolism and Diabetes
Institute of Population and Public Health

Institut de la santé des Autochtones
Institut du vieillissement
Institut du cancer
Institut de la santé circulatoire et respiratoire
Institut de la santé des femmes et des hommes
Institut de génétique
Institut des services et des politiques de la santé
Institut du développement et de la santé des enfants et des adolescents
Institut des maladies infectieuses et immunitaires
Institut de l'appareil locomoteur et de l'arthrite
Institut des neurosciences, de la santé mentale et des toxicomanies
Institut de la nutrition, du métabolisme et du diabète
Institut de la santé publique et des populations

March 15, 2014

Dr. Ari Nareg MEGUERDITCHIAN
McGill University Health Centre
Royal Victoria Hospital
687 Pine Ave. West
Room S7.30
Montreal, Québec H3A 1A1

Dear Dr. MEGUERDITCHIAN:

Congratulations on your success in the recent Canadian Institutes of Health Research funding competition.

You should take great pride in this achievement, particularly given the highly competitive nature of CIHR peer review. Excellence in research will always rest on the shoulders of individual inspiration, curiosity, and drive. Given the Canadian health research community’s global reputation for excellence, I am confident that your work will help create new scientific knowledge that will strengthen the health care system for the benefit of Canadians.

As you know, peer review is the cornerstone of our research funding system. This process is made possible because of the kind volunteerism of individuals who generously gave their time to review your application. As a CIHR-funded researcher, you may be invited to serve in the peer review process as we are continuously recruiting and retaining the most accomplished, innovative and creative scientists to review health research proposals.

To meet CIHR goals, we must also continue to communicate the value of health research to Canadians. That is why we encourage you to work with your institution to promote your research. We have developed guidelines on public communication, available at: www.cihr-irsc.gc.ca/e/30789.html, to support you in this activity.

Once again, I offer you my congratulations and best wishes for success in your research.

Yours sincerely,

Alain Beaudet, MD, Ph.D.
President

359234-201310CEH-EHI-316814-I006-168217-CONGR

President

Canadian Institutes of Health Research
Room 97, 160 Elgin Steet, Address locator: 4809A
Ottawa, (Ontario) K1A 0W9 Tel.: (613) 941-2672
Fax (613) 954-1800 **www.cihr-irsc.gc.ca**

Président

Instituts de recherche en santé du Canada
Pièce 97, 160 rue Elgin, Indice de l'adresse: 4809A
Ottawa, (Ontario) K1A 0W9 Tél.: (613) 941-2672
Fax (613) 954-1800 **www.irsc-cihr.gc.ca**

Canada

Review Type/Type d'évaluation: Committee Member 1/Membre de comité 1
Name of Applicant/Nom du chercheur: MEGUERDITCHIAN, Ari Nareg
Application No./Numéro de demande: 316814
Agency/Agence: CIHR/IRSC
Competition/Concours: 2013-10-15 Catalyst Grant: e-Health Innovations: Supporting More Efficient Population and Individualized Healthcare/Subvention catalyseur : Innovations en cybersanté : soutenir l'amélioration des soins de santé axés sur les individus et les populations
Committee/Comité: e-Health Innovations/Innovations en cybersanté
Title/Titre: Adjuvant Endocrine Therapy in Breast Cancer: A Novel E-health Approach in Optimizing Treatment Adherence in Seniors

---

Assessment/Évaluation:

Grant Title: # 316814. Electronic Health Information Tools: An Opportunity to provide Better Care to Older Breast Cancer Patients

1. Brief Synopsis of Proposal

This proposal describes a two-site comparison of an electronic adherence risk measurement, monitoring and notification program for adjuvant endrocrine therapy (AET) for HR positive breast cancer in older women in two Montreal hospitals, versus usual care over 1.5 years of follow-up.

2. Research Approach

The background makes a very good case for studying older women who start AET for breast cancer after surgery, as the therapy has been shown in meta-analysis to decrease recurrence and death.

Although the project is described as a randomized trial, there are only two sites (two clusters) involved, therefore the inherent advantages of randomization are not realized.

The intervention builds on the previous work of MOXXI, an e-health program which is now described as an e-health tool providing patient-specific decision support using real-time processing of health service claims. The level of integration with the hospital EHRs is not described. For this study, the AET adherence monitoring tool is to a) calculate 5-year treatment discontinuation risk score, b) provide an electronic alert when discontinuation risk passes and unspecified threshold, c) adherence monitoring of AET, and d) alert for presumed discontinuation.

The clinical structure, environment, current work flow, expected fit and actual personnel targets of the intervention, are not described.

The primary outcome of interest is AET discontinuation rate in the two groups with secondary outcomes examining actions taken by the intervention team, influence on workflow and costs of implementation. The secondary outcomes are very scantily described and do not include important clinical outcomes.

The authors calculate a samples size of only 86 women to detect a 40% decrease in discontinuations. Follow-up is to be 1.5 years; when taking into account recruitment, this will be at least a 2-year grant while catalyst grants are meant to be 12 months. This seems to be a *serious feasibility* problem which is not

Review Type/Type d'évaluation: Committee Member 1/Membre de comité 1
Name of Applicant/Nom du chercheur: MEGUERDITCHIAN, Ari Nareg
Application No./Numéro de demande: 316814
Agency/Agence: CIHR/IRSC
Competition/Concours: 2013-10-15 Catalyst Grant: e-Health Innovations: Supporting More Efficient Population and Individualized Healthcare/Subvention catalyseur : Innovations en cybersanté : soutenir l'amélioration des soins de santé axés sur les individus et les populations
Committee/Comité: e-Health Innovations/Innovations en cybersanté
Title/Titre: Adjuvant Endocrine Therapy in Breast Cancer: A Novel E-health Approach in Optimizing Treatment Adherence in Seniors

---

Assessment/Évaluation:

discussed.

3. Originality
The integration of provincial pharmacy administrative data with notification and decision support algorithms for clinicians, is novel.

4. End-user Requirements or Preferences Incorporated?
Not described.

5. Budget
Budget totals $265,000 with approximately $83,000 requested from CIHR. The total budget seems very large for such a small, confined project and should be reviewed. If the development budget is this large, it creates anxiety around whether timelines can be met.
Budget justification is very detailed but again suggests a larger project than is actually described. For example, why is both a part-time research assistant and a study coordinator required?

6. Applicants
The NPA is a breast cancer surgeon with research training who has been mentored within McGill University's excellent health informatics research group. His first author publication list for someone approaching Associate Professor status, is modest but grants and academic activities are good.
There are 5 other collaborators but their CVs are not included. This is a serious oversight.

7. Impact of the Research – potential to augment, integrate, evaluate e-health approaches; add value to existing service delivery; improve quality, equity, cost-effectiveness
This is a significant deficit in the grant, as important clinical outcomes are not being measured as part of this grant. Since this is a catalyst grant, the authors should have described the future large study that is planned to follow-up this pilot study.

8. Environment for Research
Appears to be excellent. Access to large secure databases is required, provided by McGill health informatics group.

9. Alignment with Funding Opportunity

**Review Type/Type d'évaluation:**	Committee Member 1/Membre de comité 1
**Name of Applicant/Nom du chercheur:**	MEGUERDITCHIAN, Ari Nareg
**Application No./Numéro de demande:**	316814
**Agency/Agence:**	CIHR/IRSC
**Competition/Concours:**	2013-10-15 Catalyst Grant: e-Health Innovations: Supporting More Efficient Population and Individualized Healthcare/Subvention catalyseur : Innovations en cybersanté : soutenir l'amélioration des soins de santé axés sur les individus et les populations
**Committee/Comité:**	e-Health Innovations/Innovations en cybersanté
**Title/Titre:**	Adjuvant Endocrine Therapy in Breast Cancer: A Novel E-health Approach in Optimizing Treatment Adherence in Seniors

Assessment/Évaluation:

Good since patient-specific decision support for an important health condition.

10. Innovative Partnerships with e-health Enterprises?

No private partnerships described.

Review Type/Type d'évaluation: Committee Member 2/Membre de comité 2
Name of Applicant/Nom du chercheur: MEGUERDITCHIAN, Ari Nareg
Application No./Numéro de demande: 316814
Agency/Agence: CIHR/IRSC
Competition/Concours: 2013-10-15 Catalyst Grant: e-Health Innovations: Supporting More Efficient Population and Individualized Healthcare/Subvention catalyseur : Innovations en cybersanté : soutenir l'amélioration des soins de santé axés sur les individus et les populations
Committee/Comité: e-Health Innovations/Innovations en cybersanté
Title/Titre: Adjuvant Endocrine Therapy in Breast Cancer: A Novel E-health Approach in Optimizing Treatment Adherence in Seniors

---

Assessment/Évaluation:

The applicants propose the use of admin claims as a method of determining adjuvant endocrine therapy (AET) on older women with breast cancer. AET has been shown to be effective, but suffers from adherence issues. A risk assessment will be generated by the system, as well as on-going monitoring and alerting of AET adherence. A trial is proposed is to evaluate the system focusing on the clinical response to the system output.

This is an excellent proposal with a novel, practical approach to determine adherence with a simple data collection method. Other methods tried have failed due to the complexity and resource intensiveness.

The applicants make a strong case for the need of such adherence support in this population; the long duration, the age of the patients, co-morbidity, the likelihood of treatment of interruption, etc. The effectiveness of AET in the population given the more limited treatment options also supports its importance.

The use of the data collection method through the MOXXI tool has been supported in other applications such as cardiovascular medications and psychoactive medications. This de-risks this proposal knowing that its been used effectively previously.

No issues with the trial methodology, research team, or budget. The trial has a significant in-kind contributions to refine the system for this application.

In summary, an excellent proposal for a novel, practical application.

Review Type/Type d'évaluation: SO Notes /Notes de l'agent scientifique
Name of Applicant/Nom du chercheur: MEGUERDITCHIAN, Ari Nareg
Application No./Numéro de demande: 316814
Agency/Agence: CIHR/IRSC
Competition/Concours: 2013-10-15 Catalyst Grant: e-Health Innovations: Supporting More Efficient Population and Individualized Healthcare/Subvention catalyseur : Innovations en cybersanté : soutenir l'amélioration des soins de santé axés sur les individus et les populations
Committee/Comité: e-Health Innovations/Innovations en cybersanté
Title/Titre: Adjuvant Endocrine Therapy in Breast Cancer: A Novel E-health Approach in Optimizing Treatment Adherence in Seniors

---

Assessment/Évaluation:

MEGUERDITCHIAN

This proposal uses a novel, practical approach to study medication adherence. The study may not examine the entire follow up period. The inherent advantages of randomization may not be realized with a cluster design. EHR integration with each hospital is not described. Some clinically important outcomes are not included in the grant. The team has done related work with this type of intervention. Commercialization opportunities may be limited.
